# Using Actigraphy to Predict the Ecological Momentary Assessment of Mood, Fatigue, and Cognition in Older Adulthood: Mixed-Methods Study

**DOI:** 10.2196/11331

**Published:** 2019-01-18

**Authors:** Carolyn M Parsey, Maureen Schmitter-Edgecombe

**Affiliations:** 1 Department of Neurology University of Washington School of Medicine Seattle, WA United States; 2 Department of Psychology Washington State University Pullman, WA United States

**Keywords:** actigraphy, aging, ecological momentary assessment, mood, sleep

## Abstract

**Background:**

Sleep quality has been associated with cognitive and mood outcomes in otherwise healthy older adults. However, most studies have evaluated sleep quality as aggregate and mean measures, rather than addressing the impact of previous night’s sleep on next-day functioning.

**Objective:**

This study aims to evaluate the ability of previous night’s sleep parameters on self-reported mood, cognition, and fatigue to understand short-term impacts of sleep quality on next-day functioning.

**Methods:**

In total, 73 cognitively healthy older adults (19 males, 54 females) completed 7 days of phone-based self-report questions, along with 24-hour actigraph data collection. We evaluated a model of previous night’s sleep parameters as predictors of mood, fatigue, and perceived thinking abilities the following day.

**Results:**

Previous night’s sleep predicted fatigue in the morning and midday, as well as sleepiness or drowsiness in the morning; however, sleep measures did not predict subjective report of mood or perceived thinking abilities the following day.

**Conclusions:**

This study suggests that objectively measured sleep quality from the previous night may not have a direct or substantial relationship with subjective reporting of cognition or mood the following day, despite frequent patient reports. Continued efforts to examine the relationship among cognition, sleep, and everyday functioning are encouraged.

## Introduction

Recent trends in behavioral health have demonstrated the importance of quality sleep in older age [1]. However, nighttime sleep disturbances are common in older adults [2] such as early waking, poorer sleep efficiency (SE), and trouble falling asleep [3-5]. Sleep difficulties in cognitively healthy older adults have been associated with self-reports of poorer physical and mental functioning [6,7], indicating the importance of sleep in successful aging (ie, the preservation of physical and cognitive functioning and avoidance of disease processes [8].

Sleep complaints in older adults are also associated with cognitive and functional difficulties [9,10]. For example, poor sleep quality has been associated with poorer global cognitive functioning [11-13], as well as with specific deficits in memory [14,15], attention, and executive functioning [16]. In addition, poorer overall health and increased daytime fatigue [17], as well as reduced participation in social and physical activities [18], have been attributed to poor sleep in the elderly. Although there is evidence that sleep problems increase in late-life, less is known about the specific sleep factors that contribute to both poorer cognitive and functional abilities. This study explored the impact of objectively measured sleep quality on self-reported measures of daily functioning (eg, mood, fatigue, and perceived cognitive functioning) in a community-dwelling older adult sample.

Ecological momentary assessment (EMA) allows the gathering of subjective measures multiple times per day [19,20]. EMA has been used extensively in research with physical activity monitoring [21,22] and to document affective changes [23,24]. A major advantage of EMA is capturing data in short timeframes, resulting in less bias from autobiographical memory strategies [25,26]. In addition, data collection occurs in the participants’ natural environment without drastically changing or influencing their daily routine [27].

Given the variability in daily experiences, EMA approaches appear ideal for the assessment of fatigue, physical activity, and fluctuations in mood during the day. Furthermore, nightly comparisons may reveal more useful information relative to aggregated or averaged data, as night-to-night variability has been associated with greater sleep complaints in the elderly [28]. Lemola et al [29] found that greater variability in the total sleep time (TST) was associated with self-report of poorer sleep quality and subjective well-being; however, average sleep duration, sleep onset latency (SOL), and wake after sleep onset (WASO) were not related to self-reported well-being. McCrae et al [30] found that lower self-reported sleep quality was associated with more negative affect, but these relationships did not achieve significance for objective sleep measures. Russell et al [31] evaluated sleep measurements as predictors of next-day fatigue in patients with chronic fatigue syndrome and found that subjective, but not objective, sleep measures predict next-day fatigue. Furthermore, they found that negative mood in the morning mediated the effect between subjective sleep and fatigue.

This exploratory study tested a model of objective sleep measures as predictors of self-report measures of cognition, mood, and fatigue at 4 time blocks the following day (ie, morning, midday, afternoon, and evening). We hypothesized that greater SOL, poorer SE, and increased WASO from the previous night would predict EMA-based reports of more negative mood, greater daytime fatigue and sleepiness or drowsiness, and poorer perceived thinking abilities the following day. These relationships were expected to be strongest in the morning and midday time blocks because of their proximity to the previous night’s sleep. In other words, if sleep quality impacts mood, cognition, or fatigue the following day, the influence would be greatest at times closest to the morning wake time (eg, feeling groggy or perception of less cognitive clarity in the morning) and prior to activities that could improve energy levels, mood, and cognition (eg, caffeine and exercise).

## Methods

### Participants

Participants aged ≥55 years were recruited from the community (eg, newspaper ads and health fairs) and completed phone interview screenings including a brief medical review, the telephone interview for cognitive status (TICS) [32], and the Modified Clinical Dementia Rating [33]. Participants were excluded from the study if they obtained a TICS score of ≤27 (the equivalent of a Mini-Mental State Examination of 24) [32] and a Modified Clinical Dementia Rating score >0, which would indicate cognitive impairment. Individuals with diagnosed sleep disorders (eg, chronic insomnia and sleep apnea) and current use of sleep medications or aids (eg, zolpidem and doxepin) were also excluded. Self-report of minor sleep complaints (eg, occasional difficulty falling asleep, staying asleep, or waking too early) were not considered exclusionary criteria, as these subthreshold sleep complaints are common in older adults and reflect normal sleep in a cognitively healthy population [3,4]. In addition, participants were screened for depression and excluded if they scored >10 on the Geriatric Depression Scale-Short Form [34], as well as other cognitive domains, including attention, verbal memory, language, and executive functioning (Table 1), to determine a cognitively healthy participant group.

In this study, 73 cognitively healthy older adults met the study criteria with, at least, 6 nights of actigraph data and <75% of EMA questions answered. The 73 participants (19 males, 54 females) had a mean age of 67.64 (SD 9.59) years. Table 1 provides the descriptive data of the sample. This study was part of a larger longitudinal study on cognition and aging; as such, all participants completed a 3-hour battery of cognitive tests and questionnaires; scores were compared with normative data, and participants whose scores fell ≥1.5 SDs below the mean were excluded from the sample (see Table 1 for average cognitive performances of the sample). After completing cognitive testing, participants wore an actigraph for 1 week while also completing EMA measures (ie, phone-based questions 4 times daily).

This study was approved for human subjects by the Washington State University Institutional Review Board under a study entitled “Activities of Daily Living, Executive Functioning and Aging” (Institutional Review Board Number 12606-011).

### Sleep Measures

#### Actigraph

Mini-Motionlogger actigraphs (Ambulatory Monitoring Inc.) were worn on the nondominant wrist for 1 week of consecutive nights. Actigraph data were collected in Proportional Integration Mode, aggregated in 60-second epochs, and analyzed using the University of California, San Diego sleep scoring algorithm [35].

The following sleep variables were used for statistical analyses:

- SOL: Time elapsed from the start of the “down” interval of nighttime sleep until the first minute scored as sleep or inactive.
- SE: Percentage of minutes scored as “sleep” within the “down” interval.
- WASO: Total minutes scored as “wake” during the “down” interval after actigraphically determined sleep onset.

**Table 1:** Demographic data and mean summary data for older adult participants.

Variable or test		Mean^a^ (SD)	Normative descriptor
**Demographics**			
	Age	67.64 (9.59)	N/A^b^
	Education (years)	16.41 (2.70)	N/A
	Gender	N/A	19 male, 54 female
**Verbal ability and global status**			
	Wechsler Test of Adult Reading total score	44.34 (3.61)	High average
	Telephone Interview for Cognitive Status total score	35.30 (2.04)	Nonimpaired
**Attention and speeded processing**			
	Symbol Digit Modalities Test Oral total	55.43 (11.84)	High average
**Verbal memory**			
	Memory Assessment Scale List Delayed Recall	11.25 (1.18)	Average
**Word finding and language**			
	Boston Naming Test total correct	57.23 (2.96)	High average
**Executive functioning**			
	D-KEFS^c^ Letter Fluency	41.90 (11.84)	Average
	D-KEFS Design Fluency	26.62 (7.04)	Average
	Frontal Assessment Battery total	16.83 (1.68)	Nonimpaired

^a^Unless otherwise indicated, mean scores are raw scores.

^b^N/A: not applicable.

^c^D-KEFS: Delis-Kaplan Executive Functioning System.

**Ecological momentary assessment phone questions and response options.**

"Your general thinking abilities are currently...”

- Response options: Very Good, Good, Fair, Poor, Very Poor (1-5, respectively)

"Your general mood is currently...”

- Response options: Very Good, Good, Fair, Poor, Very Poor (1-5, respectively)

"How fatigued do you feel currently?"

- Response options: Not at all or none, A Little Bit, Somewhat, Quite a Bit, Very Much (1-5, respectively)

"In the past 2 hours, how sleepy or drowsy have you felt?"

- Response options: Not at all or none, A Little Bit, Somewhat, Quite a Bit, Very Much (1-5, respectively)

#### Ecological Momentary Assessment

EMA self-report measures of mood, fatigue, sleepiness or drowsiness, and perceived thinking were obtained using an automated phone system for 7 consecutive days (corresponding to actigraph data collection). Each day was divided into 4 time blocks as follows: morning (9:30-11:30 am); midday (12:30-2:30 pm); afternoon (3:30-5:30 pm); and evening (6:30-8:30 pm). Participants received an automated call at a random time during each time block. If they did not answer the phone, the system automatically redialed 10 minutes later (up to 2 redials within each block). The same 4 questions were asked at each time block, including the current assessment of mood, fatigue, drowsiness, and thinking abilities (Textbox 1). Questions included a 2-hour time window, “In the past 2 hours...,” to capture the time elapsed within the 2-hour time block. Participants used the numeric phone keypad to respond to questions using Likert-style continuums (eg, “For ‘Very Good’, press 1”).

The average TST for the sample was 413.69 (SD 76.60) minutes, which equates to roughly 6.89 hours of sleep per night. However, Spearman correlations revealed that the TST and the EMA question of daily activity completion did not demonstrate correlations with any other variable (ie, correlations >.200); thus, actigraphic TST and EMA completion of daily activities were not included in regression analyses. For comprehensiveness, when ordinal logistic regression (LR) models were run with and without TST, the presence of TST did not influence the outcome of the model. To increase the power of the ordinal logistic regression models, as well as eliminate predictor variables that did not demonstrate preexisting relationships with dependent variables, the TST was not included as a predictor of the EMA data in the regression models.

All variables were evaluated for normality prior to conducting statistical analyses. Although the EMA data were skewed, the transformation of the EMA data would make it difficult to interpret findings of the ordinal logistic regression models. Rather than using transformation techniques, and to preserve the ordinal nature of the EMA data, statistical procedures were selected depending on the data type. Spearman correlations (ρ) were conducted for rank-order correlations that did not assume a normal distribution (eg, EMA questions), while Pearson correlations (*r*) were conducted for actigraph data, which were normally distributed. Initial correlations were conducted to identify relationships between EMA and actigraph data. Then, a within-subjects ordinal logistic regression model was run using the variables that surfaced as having significant relationships with the dependent measure (per findings of Spearman correlations at *P*<.01); this model evaluated the influence of previous night’s sleep measures on the EMA data the following day.

Ordinal logistic regression models were run individually for the prediction of the EMA data at each time block. Participants’ age was held constant in all models. Measures of SOL, SE, and WASO from the previous night’s sleep were entered simultaneously as predictors of EMA self-reports of mood, fatigue, sleepiness or drowsiness, and perceived thinking abilities at morning, midday, afternoon, and evening time blocks the following day. Significance values for model fit were set at *P*<.01.

## Results

### Actigraph Sleep Data

Participants wore actigraphs for an average of 7.47 nights (SD 0.40). Measures of SE (mean 91.93% [SD 5.02]), SOL (mean 20.30 [SD 16.23] minutes), and WASO (mean 38.10 [SD 28.34] minutes) were consistent with cognitively healthy older adult samples in other studies [36]. Longer SOL (*r*=−.361, *P*=.002), but not SE and WASO (*r*=−.096 to.020, *P=*.002), correlated with older age. Table 2 presents actigraph data for the participant sample.

### Ecological Momentary Assessment Data

**Figure 1:**
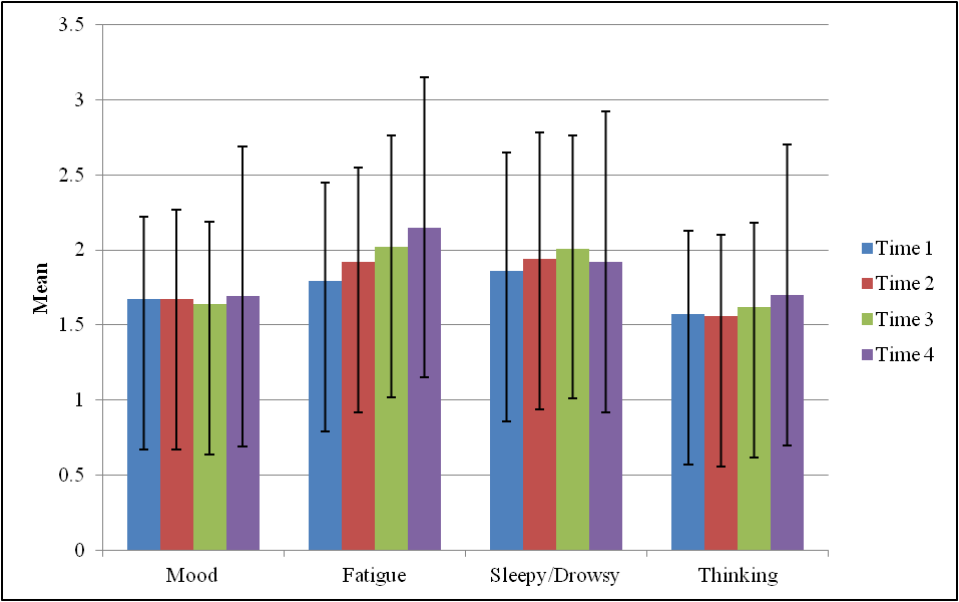
Mean values of ecological momentary assessment variables across times 1-4.

On average, participants completed EMA questions for 7.96 (SD 0.44) days and answered an average of 79.52% morning, 75.35% midday, 81.49% afternoon, and 83.03% evening phone calls. Older age exhibited small correlations with EMA reports of greater sleepiness or drowsiness at the morning time block (ρ=−.265, *P*=.03) and more negative mood at the morning time block (ρ=−.238, *P*=.04). Figure 1 shows the mean values of the EMA data.

### Spearman Correlations

Correlations of evening EMA data (Day A, Time 4) with the EMA data the following day (Day B, Times 1-4) revealed that prior evening self-reports of mood, fatigue, sleepiness or drowsiness, and perceived thinking abilities generally correlated with EMA reports for identical questions the next morning, midday, afternoon, and evening (ρ=.389-.747, *P*=.002-.001; Table 3). All EMA questions generally correlated with each other at all time blocks (ρ=.335-.794, *P*=.001), except mood and perceived thinking abilities with fatigue and sleepiness or drowsiness at the evening time block (Table 3).

### Model of Actigraphy and Ecological Momentary Assessment Data

#### Relationships Between Actigraph and Ecological Momentary Assessment Data

Spearman correlations (Table 4) revealed that greater WASO and poorer SE were related to EMA reports of greater fatigue (WASO: ρ=.395, *P*=.005; SE: ρ=−.402, *P*=.004) and greater sleepiness or drowsiness (WASO: ρ=.381, *P*=.01; SE: ρ=−.404, *P=*.004) at the morning EMA time block the following day. In addition, longer SOL from the previous night correlated significantly with greater fatigue at the afternoon time block (ρ=.372, *P=*.01). None of the other sleep variables from the previous night correlated significantly with any of the EMA questions at midday (ρ=−.358 to.347, *P*=.002-.003) or evening time blocks (ρ=−.292 to.239, *P*=.008-.009) the following day.

#### Ordinal Logistic Regression Analyses

##### Sleep Predicting Ecological Momentary Assessment Report of Mood

The model did not indicate adequate fit for the morning (LR *χ*^2^_3_=3.68, *P*=.30), midday (LR *χ*^2^_3_=2.37, *P*=.50), afternoon (LR *χ*^2^_3_=6.14, *P*=.11), or evening (LR *χ*^2^_3_=2.77, *P*=.43) time blocks when predicting mood. None of the sleep measures emerged as significant predictors of EMA reports of mood at any of the 4 time blocks the following day (*z*=0.24-0.74, *P*>.05).

##### Sleep Predicting Ecological Momentary Assessment Report of Fatigue

When predicting fatigue the next morning (Time 1), the model showed adequate fit (LR *χ*^2^_3_=8.05, *P*=.04). Regression coefficients for sleep predictors indicated that decreased SE (odds ratio, OR, 1.16, 95% CI 1.08-1.28) predicted an increase in fatigue in the morning time block, but WASO (OR 1.07, 95% CI 0.96-1.14) and SOL (OR 1.01, 95% CI 0.98-1.04) did not (Table 5).

When previous night’s sleep measures were used to predict fatigue at midday the following day, the model demonstrated adequate fit (LR *χ*^2^_3_=11.49, *P*=.004). Evaluation of regression coefficients indicated that an increase in SE (OR 1.12, 95% CI 0.73-1.20) predicted a decrease in the EMA-based report of fatigue. Furthermore, WASO (OR 1.05, 95% CI 0.97-1.11) and SOL (OR 1.00, 95% CI 0.97-1.03) did not predict significant changes in EMA-based report of fatigue.

**Table 2:** Participant averages of actigraph variables and correlations with participants’ age.

Actigraph	Mean (SD)	Pearson’s *r*	*P* value
Sleep onset latency (min)	20.33 (16.23)	−.361^a^	.002
Sleep efficiency (%)	91.93 (5.02)	.202	.08
Wake after sleep onset (min)	38.18 (28.34)	−.193	.10

^a^Significant at *P*<.01.

The models of actigraph sleep parameters did not indicate adequate fit for predicting EMA reports of fatigue in the afternoon (LR *χ*^2^_3_=5.85, *P*=.12) or evening (LR *χ*^2^_3_=2.28, *P*=.52). Sleep parameters from the previous night did not predict changes in EMA reports of fatigue the following afternoon or evening (*z*=−0.62 to 0.29, *P*>.05).

##### Sleep Predicting Ecological Momentary Assessment Report of Sleepiness or Drowsiness

When SOL, WASO, and SE from the previous night were used as predictors of EMA report of sleepiness or drowsiness the next morning, the ordinal logistic regression model showed adequate fit (LR *χ*^2^_3_=15.06, *P*=.002). Regression coefficients indicated that increased SE predicted a decrease in the EMA-based report of sleepiness or drowsiness the following morning (OR 0.87, 95% CI 0.63-1.06). However, WASO (OR 0.99, 95% CI 0.94-1.04) and SOL (OR 1.02, 95% CI 0.98-1.04) did not predict significant changes in EMA-based report of sleepiness or drowsiness.

Furthermore, models of sleep variables predicting EMA reports of sleepiness or drowsiness did not indicate adequate fit for midday (LR *χ*^2^_3_=6.59, *P*=.09), afternoon (LR *χ*^2^_3_=3.94, *P*=.27), or evening (LR *χ*^2^_3_=5.70, *P*=.13) time blocks. As such, sleep parameters from the previous night did not predict changes in EMA reports of sleepiness or drowsiness at midday, afternoon, or evening time blocks the following day (*z*=−1.22 to −0.09, *P*>.05).

##### Sleep Predicting Ecological Momentary Assessment Report of Perceived Thinking Abilities

When SOL, WASO, and SE from the previous night were used as predictors of EMA report of perceived thinking abilities the next morning, the ordinal logistic regression models did not demonstrate adequate fit for any of the EMA time blocks, including morning (LR *χ*^2^_3_=5.80, *P*=.12), midday (LR *χ*^2^_3_=1.70, *P*=.64), afternoon (LR *χ*^2^_3_=0.27, *P*=.96), or evening (LR *χ*^2^_3_=0.97, *P*=.81). As such, none of the sleep parameters emerged as significant predictors of EMA reports of perceived thinking abilities the following day (*z*=−1.73 to 0.26, *P*>.05).

**Table 3:** Spearman correlations between previous night and ecological momentary assessment questions the following morning, midday, afternoon, and evening.

Day B		Day A, Evening			
		Mood	Fatigue	Sleepy or drowsy	Perceived thinking abilities
**Morning**					
	Mood	.747^a^	.541^a^	.419^a^	.794^a^
	Fatigue	.535^a^	.579^a^	.335^a^	.465^a^
	Sleepy or drowsy	.426^a^	.507^a^	.389^a^	.417^a^
	Perceived thinking abilities	.610^a^	.479^a^	.386^a^	.674^a^
**Midday**					
	Mood	.777^a^	.507^a^	.358^a^	.726^a^
	Fatigue	.458^a^	.655^a^	.383^a^	.426^a^
	Sleepy or drowsy	.374^a^	.529^a^	.478^a^	.393^a^
	Perceived thinking abilities	.606^a^	.506^a^	.353^a^	.655^a^
**Afternoon**					
	Mood	.675^a^	.591^a^	.451^a^	.675^a^
	Fatigue	.636^a^	.720^a^	.453^a^	.571^a^
	Sleepy or drowsy	.453^a^	.409^a^	.562^a^	.419^a^
	Perceived thinking abilities	.711^a^	.554^a^	.437^a^	.771^a^
**Evening**					
	Mood	.474^a^	.437^a^	.430^a^	.648^a^
	Fatigue	.284	.531^a^	.571^a^	.305
	Sleepy or drowsy	.197	.218	.560^a^	.194
	Perceived thinking abilities	.383^a^	.420^a^	.359	.517^a^

^a^Significant correlation at *P*<.01.

**Table 4:** Spearman correlations of ecological momentary assessment variables with previous night’s sleep data.

Ecological momentary assessment (next day)		Sleep onset latency (min)	Sleep efficiency (%)	Wake after sleep onset (min)
**Morning (Time 1)**				
	Mood	.208	−.287	.284
	Fatigue	.258	−.402	.395^a^
	Sleepy or drowsy	.273	−.404^a^	.381^a^
	Perceived thinking abilities	.174	−.327	.304
**Midday (Time 2)**				
	Mood	.262	−.144	.096
	Fatigue	.328	−.358	.347
	Sleepy or drowsy	.257	−.316	.267
	Perceived thinking abilities	.312	−.232	.166
**Afternoon (Time 3)**				
	Mood	.204	−.227	.192
	Fatigue	.372^a^	−.329	.287
	Sleepy or drowsy	.208	−.228	.163
	Perceived thinking abilities	.255	−.153	.041
**Evening (Time 4)**				
	Mood	.197	−.262	.165
	Fatigue	.229	−.273	.225
	Sleepy or drowsy	.145	−.292	.239
	Perceived thinking abilities	.229	−.133	.054

^a^Significant correlation at *P*<.01.

**Table 5:** Ordinal logistic regression odds ratios for models predicting ecological momentary assessment self-reports based on sleep variables obtained the previous night.

Ecological momentary assessment (next day)		Actigraph data (Previous night)			χ^2^	*P* value
		Sleep efficiency	Sleep onset latency	Wake after sleep onset		
**Morning (Time 1)**						
	Mood	1.02	1.02	1.04	3.68	.30
	Fatigue	1.16	1.01	1.07	8.05^a^	.04
	Sleepy or drowsy	0.87	1.02	0.99	15.06^a^	.002
	Perceived thinking abilities	0.87	1.03	0.99	2.80	.12
**Midday (Time 2)**						
	Mood	1.11	1.00	1.05	2.37	.50
	Fatigue	1.12	1.00	1.05	11.49^a^	.01
	Sleepy or drowsy	1.00	1.01	1.02	6.59	.09
	Perceived thinking abilities	0.99	1.01	1.02	1.70	.63
**Afternoon (Time 3)**						
	Mood	1.05	0.99	1.04	6.14	.11
	Fatigue	0.93	1.01	1.02	5.85	.12
	Sleepy or drowsy	0.86	1.00	0.99	3.94	.27
	Perceived thinking abilities	0.84	1.00	0.99	.27	.97
**Evening (Time 4)**						
	Mood	1.01	1.00	1.02	2.77	.42
	Fatigue	1.20	1.00	1.04	2.28	.52
	Sleepy or drowsy	1.01	1.00	1.01	5.70	.13
	Perceived thinking abilities	0.97	1.00	0.99	.97	.81

^a^Significant at *P*<.05. Confidence intervals are reported in-text.

## Discussion

### Principal Findings

This study was an exploratory approach to examine relationships between objectively measured sleep quality (eg, WASO, SOL, and SE) and self-report of mood, fatigue, sleepiness or drowsiness, and perceived thinking abilities in a cognitively healthy older adult sample using actigraphy and EMA phone-based self-reports.

Older age was related to EMA reports of greater sleepiness or drowsiness and negative mood in the morning. Relationships between participants’ age and EMA questions of fatigue and perceived thinking abilities did not achieve significance. When the EMA data were compared between the previous night and the following day, reports of fatigue were markedly lower in the morning; however, EMA reports of sleepiness or drowsiness remained stable from the evening to morning and midday time blocks the following day. It is possible that the EMA questions of fatigue and sleepiness or drowsiness are not measuring identical constructs and that the wording of these 2 questions affected the reporting by participants (ie, “current” vs “past 2 hours”).

Consistent with the initial hypotheses, EMA reports of fatigue and sleepiness or drowsiness were related to previous night’s sleep. Specifically, poorer SE was related to greater sleepiness or drowsiness the next morning. However, WASO and SOL were not significant predictors of EMA measures of sleepiness or drowsiness and fatigue the following morning. Furthermore, the TST was removed from the regression analyses owing to a limited relationship with any of the dependent measures, and no evidence of contributing to the overall model. Sleep measures were not strongly related to mood and perceived thinking abilities; this relationship was likely affected by the minimal variation in the EMA data for these questions (ie, mostly average reports).

Findings of predictive relationships between sleep the previous night and EMA measures the next morning were inconsistent. Poorer SE was associated with increased levels of sleepiness or drowsiness at the morning time block and levels of fatigue at the morning and midday time blocks. These findings support the research of McCrae et al [17] who found relationships between greater self-reported sleep problems with subjective complaints of daytime fatigue in older adults. This study expands these findings to include an objective assessment of sleep (actigraphy) as predictors of subjective daytime fatigue.

Previous night’s sleep predicted only morning or midday EMA reports but did not predict EMA reports of fatigue and sleepiness or drowsiness at afternoon and evening time blocks. Other daytime factors (eg, naps and consumption of caffeine) may mitigate the impact of the previous night’s sleep on energy levels later in the day. For example, one study found that greater variability in daytime naps was associated with poorer health status [37], and it is recommended that future research consider the implication of naps on cognitive and functional abilities.

Previous night’s sleep did not predict EMA reports of mood and perceived thinking abilities the next day. Although the literature suggests that self-reported poor sleep is associated with depression and decreased functional status [38], this study found that objective sleep was not directly associated with mood. Our findings support the research of previous studies [30] that subjective, but not objective, sleep quality was associated with self-reported affect (eg, poorer sleep quality correlated with more negative affect). Of note, participants in this study were screened for depressive symptoms at the outset; thus, relationships with mood may be affected by the baseline levels of emotional symptoms for the participants. Interestingly, our results contrast the findings of Russell et al [31] who found that subjective but not objective sleep measures predicted self-reported fatigue the following morning, as our objective sleep measures did predict reports of next-morning fatigue; however, this study was in a generally healthy older adult sample, whereas others evaluated patients with chronic fatigue syndrome [31], suggesting differences in detections within clinical populations.

### Limitations

The study sample was predominantly female, highly educated, and racially homogenous, which may limit the generalization of findings to other demographic groups. The number of participants excluded from the initial sample was substantial as a result of study requirements; larger sample sizes, as well as comparisons of healthy groups to those with sleep disorders or sleep medications, would be useful to assess the influence of medical and pharmaceutical impact on sleep and daily functioning. By nature of the EMA data, there were many individual variables and, as a result, a large number of analyses were conducted in this study, increasing the possibility of false-positive findings. To compensate for this, we used more stringent *P* values and reduced variables in the models to only those identified in initial correlation analyses (ie, removal of actigraphic TST and EMA daily activity performance). Regarding EMA limitations, the type of telephone number (eg, home vs cell phone) may have influenced the data collection, such that individuals who provided home phone numbers may not have been home to respond to the phone calls; the qualitative nature of missed response items is worthy of further investigation. In addition, the time blocks chosen for this study were based on focus-group information; however, this may not have accurately captured variation in wake-up time (ie, participants who would normally wake up sooner or later than the phone call period). Sensor-based assessment, such as using actigraphy or wrist-based fitness devices, could be a way to prompt EMA questions within a designated time of waking up, thus adjusting to the individual chronology of participants.

### Future Directions

Future research should consider longitudinal effects of sleep and perceived functioning on actual cognitive and everyday performance. For example, if maintaining consistent sleep patterns is found to be more predictive of perceived functioning and, thus, contributing to actual cognitive performance, this could inform treatments for sleep disruption. Given the prevalence of naps as individuals age [39], it would be beneficial to include the influence of napping in models of sleep and daily functioning (eg, pre- and postnap EMA reports). Furthermore, research on sleep and neurodegenerative disease has explored the sleep profiles of those with mild cognitive impairment [40,41] and dementia [42], as well as neurological changes in poor sleepers with cognitive deficits [43]. Additional research efforts should investigate whether poor sleep is a robust contributor or risk factor for cognitive decline, as monitoring changes in sleep could be beneficial for the treatment of neurodegenerative disease.

## Abbreviations

D-KEFS: Delis-Kaplan Executive Functioning System
EMA: ecological momentary assessment
LR: logistic regression
SE: sleep efficiency
SOL: sleep onset latency
TICS: telephone interview for cognitive status
TST: total sleep time
WASO: wake after sleep onset
